# Chironomid association with *Vibrio cholerae*

**DOI:** 10.1128/spectrum.02567-23

**Published:** 2023-12-07

**Authors:** Malka Halpern

**Affiliations:** 1 Department of Biology and Environment, University of Haifa at Oranim, Tivon, Israel; 2 Department of Evolutionary and Environmental Biology, University of Haifa, Haifa, Israel; McGill University, Sainte-Anne-de-Bellevue, Quebec, Canada

**Keywords:** *Vibrio cholerae*, chironomid, larvae, egg mass

## LETTER

Zhao et al. (1) created freshwater microcosms to examine the impact of *Vibrio cholerae* cell density in the water on chironomid larvae. In their work, they stated that nothing is known about the dynamics and the interactions between *V. cholerae* and chironomids. However, this is not the case as 22 years ago, we (2), were the first to describe an association between chironomids and *V. cholerae*. We were also the first to demonstrate that all four chironomid life stages act as *V. cholerae* natural reservoirs (2 – 12).

We have shown that *V. cholerae* produces the extracellular enzyme hemagglutinin/protease (HAP), which can degrade chironomid egg masses and prevent eggs from hatching (13). In a yearly field survey, we found that when chironomid populations are low, the abundance of *V. cholerae* in the egg masses is also low [less than 5 × 10^1^ colony forming units (CFU)/egg mass] (6, 14). However, when chironomid populations peak, *V. cholerae* numbers in the egg masses rise by an order of magnitude (5 × 10^2^ CFU/egg mass), and HAP is produced, causing egg mass degradation. As a result, larvae do not hatch, and the chironomid population declines. Thus, the interactions between *V. cholerae* and chironomid populations in aquatic environments can be defined as a prey-predator dynamics as HAP plays a role in controlling the number of chironomids in the environment (5, 7, 13, 14).

**Fig 1:**
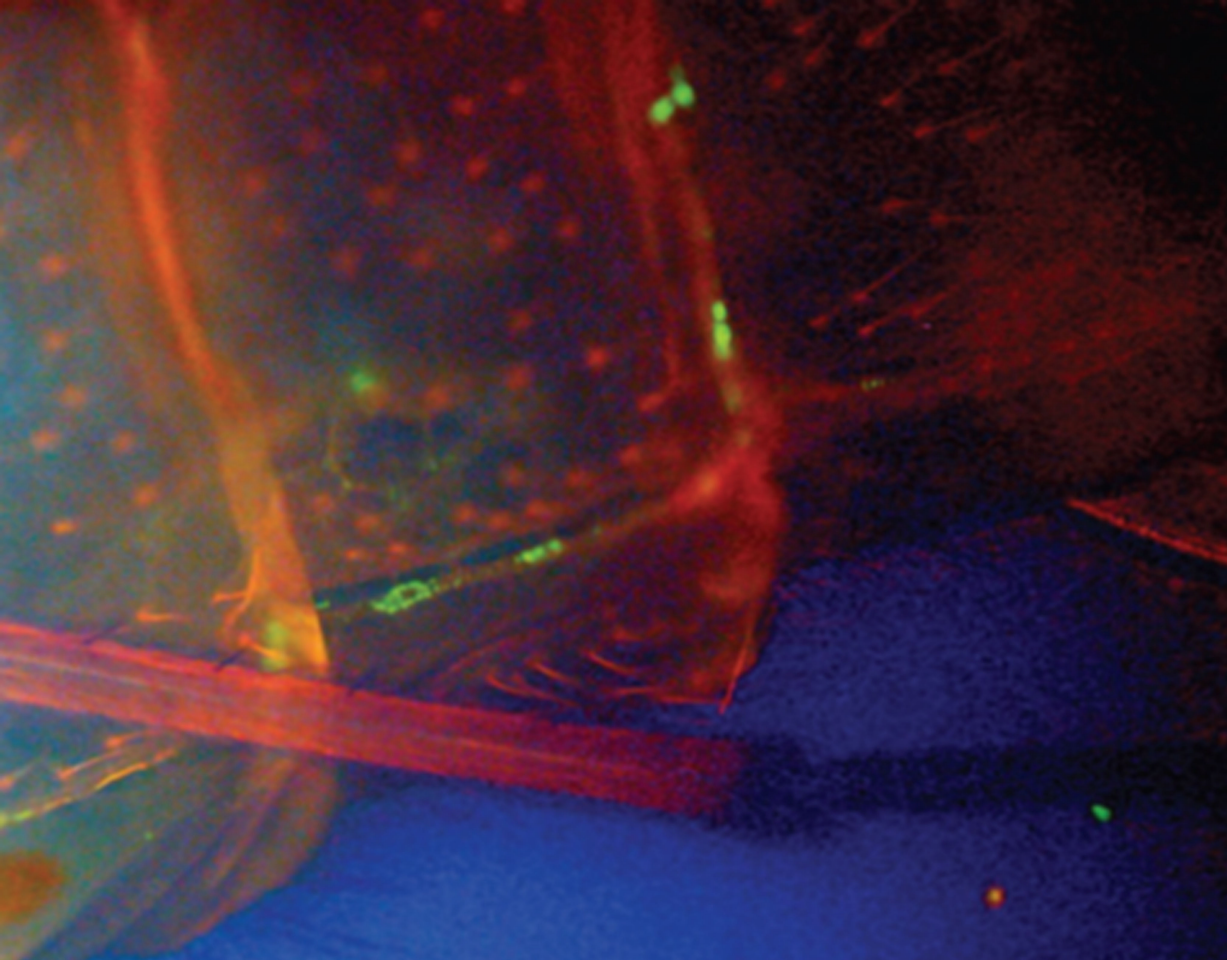
*Vibrio cholerae* tagged with GFP, located on the intersegmental membrane of an adult chironomid exoskeleton. The adult chironomid emerged from a bottle in which chironomid larvae were incubated with *V. cholerae* serogroups O9, O1, or O139 tagged with GFP. The chironomid was examined under an epifluorescent microscope. Here, a dorsal view of three abdominal segments is shown. Adopted with permission from reference (4).

In a laboratory culture, we demonstrated that toxigenic *V. cholerae* O1 and O139 strains marked with green fluorescent protein (GFP) can be transferred from water to larvae, from larvae to adults, and from one laboratory flask to another (through flying adults) (Fig. 1) (4). This phenomenon was also confirmed in a field experiment when the transfer of *V. cholerae* by adult chironomids from a waste stabilization pond to bacteria-free water pools was demonstrated (4).

Zhao et al. (1) found that *V. cholerae* in the concentration of 10^9^ CFU/mL caused larval death. At this bacterial load, any bacterial species will cause larval death because of the high organic load and lack of oxygen. Freshwater microcosm experiments should use lower bacterial concentrations as was demonstrated by Broza et al. (4) who introduced 10^6^ CFU/mL GFP-tagged bacteria into a laboratory flask containing larvae to demonstrate *V. cholerae* transfer to pupae and adults (Fig. 1). In this setting, no larval death was recorded. Moreover, *V. cholerae* in a concentration of 10^3^ CFU/mL (or less) was used to demonstrate that *V. cholerae* can proliferate on chironomid egg masses as a sole carbon source (2, 15). The numbers of the bacterial CFU increased by several orders of magnitude, reaching 10^6^ CFU/mL, which is the maximal bacterial load that should be used in freshwater microcosm experiments (2, 15).

To conclude, chironomids are natural reservoirs of *V. cholerae,* and indeed, their interactions should be studied to provide better insights into the devastating human pathogen, *V. cholerae* (16). However, the experimental conditions should mimic the natural environmental conditions to allow for broad inference (4, 6, 15).
